# 2,6-Diethyl­anilinium dihydrogen phosphate–phospho­ric acid (1/1)

**DOI:** 10.1107/S1600536810051159

**Published:** 2010-12-11

**Authors:** Hamed Khemiri, Samah Toumi Akriche, Salem S. Al-Deyab, Mohamed Rzaigui

**Affiliations:** a Laboratoire de Chimie des Matériaux, Faculté des Sciences de Bizerte, 7021 Zarzouna Bizerte, Tunisia; b Petrochemical Research Chair, College of Science, King Saud University Ryadh, Saudi Arabia

## Abstract

In the crystal structure of the title salt, C_10_H_16_N^+^·H_2_PO_4_^−^·H_3_PO_4_, the H_2_PO_4_^−^ and H_3_PO_4_ components are connected into infinite chains extending along the *b*-axis direction by way of O—H⋯O links. These chains are also linked through O—H⋯O hydrogen bonds thus building up a supra­molecular two-dimensional framework extending parallel to (001). The organic cations cross-link the anionic layers by way of multiple N—H⋯O inter­actions, leading to a cohesive network.

## Related literature

For hydrogen bonds, see: Blessing (1986 ▶); Desiraju (1995 ▶). For their biological occurence, see: Richards *et al.* (1972 ▶); Perutz & Ten Eyck (1972 ▶). For related structures with phosphoric acid, see: Belam *et al.* (2005 ▶); Mighell *et al.* (1969 ▶); Smith *et al.* (1955 ▶). For related organic cations, see: Akriche & Rzaigui (2008 ▶); Smirani Sta *et al.* (2010 ▶).
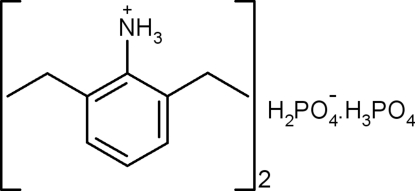


## Experimental

### 

#### Crystal data

C_10_H_16_N^+^·H_2_O_4_P^−^·H_3_O_4_P
*M* *_r_* = 345.22
Monoclinic, 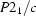

*a* = 8.1634 (10) Å
*b* = 7.707 (2) Å
*c* = 25.680 (6) Å
β = 102.686 (19)°
*V* = 1576.2 (6) Å^3^
*Z* = 4
Mo *K*α radiation
μ = 0.31 mm^−1^
*T* = 293 K
0.45 × 0.30 × 0.20 mm

#### Data collection

Enraf–Nonius TurboCAD-4 diffractometer
5173 measured reflections
2776 independent reflections
2417 reflections with *I* > 2σ(*I*)
*R*_int_ = 0.011
2 standard reflections every 120 min  intensity decay: 4%

#### Refinement

*R*[*F* ^2^ > 2σ(*F* ^2^)] = 0.036
*wR*(*F* ^2^) = 0.104
*S* = 1.06
2776 reflections
198 parameters
H-atom parameters constrained
Δρ_max_ = 0.27 e Å^−3^
Δρ_min_ = −0.47 e Å^−3^

### 

Data collection: *CAD-4 EXPRESS* (Enraf–Nonius, 1994 ▶); cell refinement: *CAD-4 EXPRESS*; data reduction: *XCAD4* (Harms & Wocadlo, 1996 ▶); program(s) used to solve structure: *SHELXS97* (Sheldrick, 2008 ▶); program(s) used to refine structure: *SHELXL97* (Sheldrick, 2008 ▶); molecular graphics: *ORTEPIII* (Burnett & Johnson, 1996 ▶), *ORTEP-3 for Windows* (Farrugia, 1997 ▶) and *DIAMOND* (Brandenburg & Putz, 2005 ▶); software used to prepare material for publication: *WinGX* (Farrugia, 1999 ▶).

## Supplementary Material

Crystal structure: contains datablocks I, global. DOI: 10.1107/S1600536810051159/dn2632sup1.cif

Structure factors: contains datablocks I. DOI: 10.1107/S1600536810051159/dn2632Isup2.hkl

Additional supplementary materials:  crystallographic information; 3D view; checkCIF report

## Figures and Tables

**Table 1:** Hydrogen-bond geometry (Å, °)

*D*—H⋯*A*	*D*—H	H⋯*A*	*D*⋯*A*	*D*—H⋯*A*
O2—H2⋯O1^i^	0.82	1.84	2.540 (2)	142
O3—H3⋯O8^ii^	0.82	1.72	2.520 (2)	166
O4—H4⋯O7^iii^	0.82	1.74	2.521 (2)	158
O5—H5⋯O1	0.82	1.86	2.664 (2)	165
O6—H6⋯O7^ii^	0.82	1.76	2.577 (2)	171
N1—H1*A*⋯O6	0.89	2.18	2.927 (2)	141
N1—H1*B*⋯O8^iv^	0.89	1.89	2.772 (2)	172
N1—H1*C*⋯O2	0.89	1.98	2.861 (3)	168

Symmetry codes: (i) 
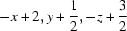
; (ii) 
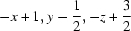
; (iii) 
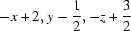
; (iv) 
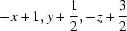
.

**Fig. 1.**
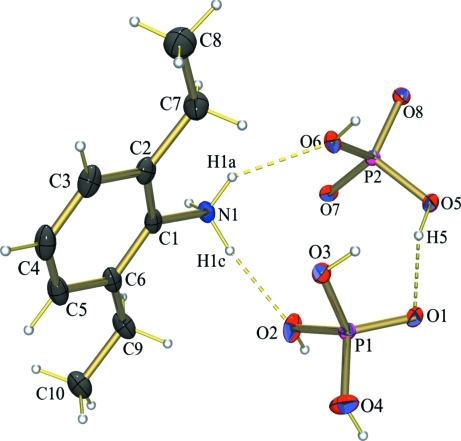
View of (I) with the atom-labelling scheme. Displacement ellipsoids are drawn at the 30% probability level. H atoms are represented as small spheres of arbitrary radii. Hydrogen bonds are represented as dashed lines.

**Fig. 2.**
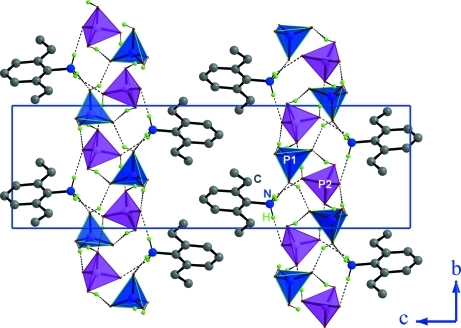
Projection of (I) along the a axis. The H-atoms not involved in H-bonding are omitted. H bonds are shown as dashed lines.

## supplementary crystallographic information

### Comment

The H_3_PO_4_ molecules play an important role for structure cohesion of adduct materials, by connecting the other components of crystal by a network of hydrogen bonds particularly strong (Desiraju, 1995; Blessing, 1986). Such hydrogen bonds are of interest because of their widespread biological occurrence. For example, hydrogen bonds between phosphate groups and histidyl, imidazolyl groups are involved in the active-site substrate-binding mechanism of ribonuclease (Richards *et al.*, 1972) and in the regulation of the oxygen affinity of deoxyhemoglobin by 2,3-diphosphoglycerate (Perutz *et al.*, 1972). The influence of the hydrogen bond scheme on the building of supramolecular anionic packing for the title compound is discussed with regard to other adduct phosphates.

The asymmetric unit of (I) consists of two phosphoric entities (H_2_PO_4_^-^ and H_3_PO_4_) and one 2,6-diethylanilinium organic cation (Fig. 1). A view of the structure projected along the *b* direction (Fig. 2) shows that inorganic layers are built by H_2_PO_4_^-^ anions and H_3_PO_4_ molecules. Via O2—H2···O1 and O6—H6···O7 intermolecular hydrogen bonds, each phosphoric entity form a corrugated chain extending along the *b*-axis. These undulating chains are further linked through O3—H3···O8, O4—H4···O7 and O5—H5···O1 hydrogen bonds with short distances varying between 1.72 and 1.86 Å (Table 1), thus building up an extended supramolecular two-dimensional framework parallel to the *ab* plane. This topology is slightly different from that of (C_6_H_14_N)H_2_PO_4_H_3_PO_4_ (Belam *et al.*, 2005) adduct one, where the H_2_PO_4_^-^ anions and H_3_PO_4_ molecules are connected alternatively by O—H···O hydrogen bonds to form infinite corrugated chains parallel to the *c* direction.

It is also useful to compare the geometrical parameters of phosphoric entities in the title compound and in the crystallized H_3_PO_4_ (Mighell *et al.*, 1969; Smith *et al.*, 1955). This comparison does not show significant differences that could be generated by the organic molecule in the provided geometric properties of these species. The 2,6-diethylanilinium cations are pendant on both the faces of the two-dimensional inorganic sheet by establishing intermolecular N—H···O hydrogen bonds thanks to the NH_3_^+^ group. Among the three hydrogen atoms of the ammonium group only one, establishes a hydrogen bond with the H_3_PO_4_ molecule. The remaining ones are connected to oxygen atoms of H_2_PO_4_^-^ anions.

The geometrical hydrogen bonding scheme are given in Table 1. Examination of geometrical features of the organic entity shows that bond lengths and angles exhibit no deviations from the usually values observed in others related 2,6-diethylanilinium structures (C_10_H_16_N)ClO_4_ (Smirani Sta *et al.*, 2010) and (C_10_H_16_N)_2_H_2_P_2_O_7_.2H_2_O (Akriche *et al.*, 2008).

### Experimental

A solution of orthophosphoric acid (0,50 mmol in 30 ml of water) was added drop by drop to an ethanolic solution (5 ml) of 2,6-diethylaniline (2,488 mmol in 5 ml of ethanol). The so-obtained solution is kept in a sealed tube for three days and then submitted to a slow evaporation until the formation of good quality crystals, stable under normal conditions of temperature and moisture.

### Refinement

All H atoms attached to C, N and O atoms were fixed geometrically and treated as riding with C—H = 0.93 Å, N—H= 0.86 Å and O—H = 0.86 Å with *U*_iso_(H) = 1.2 *U*_eq_(C, N or O).

### Crystal data

**Table:**

C_10_H_16_N^+^·H_2_O_4_P^−^·H_3_O_4_P	*F*(000) = 728
*M**_r_* = 345.22	*D*_x_ = 1.455 Mg m^−^^3^
Monoclinic, *P*2_1_/*c*	Mo *K*α radiation, λ = 0.71073 Å
Hall symbol: -P 2ybc	Cell parameters from 25 reflections
*a* = 8.1634 (10) Å	θ = 9–11°
*b* = 7.707 (2) Å	µ = 0.31 mm^−^^1^
*c* = 25.680 (6) Å	*T* = 293 K
β = 102.686 (19)°	Prism, colourless
*V* = 1576.2 (6) Å^3^	0.45 × 0.30 × 0.20 mm
*Z* = 4	

### Data collection

**Table:**

Enraf–Nonius TurboCAD-4 diffractometer	*R*_int_ = 0.011
Radiation source: fine-focus sealed tube	θ_max_ = 25.0°, θ_min_ = 2.6°
graphite	*h* = −9→9
non–profiled ω scans	*k* = −9→0
5173 measured reflections	*l* = −30→26
2776 independent reflections	2 standard reflections every 120 min
2417 reflections with *I* > 2σ(*I*)	intensity decay: 4%

### Refinement

**Table:**

Refinement on *F*^2^	Primary atom site location: structure-invariant direct methods
Least-squares matrix: full	Secondary atom site location: difference Fourier map
*R*[*F*^2^ > 2σ(*F*^2^)] = 0.036	Hydrogen site location: inferred from neighbouring sites
*wR*(*F*^2^) = 0.104	H-atom parameters constrained
*S* = 1.06	*w* = 1/[σ^2^(*F*_o_^2^) + (0.0631*P*)^2^ + 0.6675*P*] where *P* = (*F*_o_^2^ + 2*F*_c_^2^)/3
2776 reflections	(Δ/σ)_max_ = 0.001
198 parameters	Δρ_max_ = 0.27 e Å^−^^3^
0 restraints	Δρ_min_ = −0.47 e Å^−^^3^

### Special details

**Table:**

Geometry. All e.s.d.'s (except the e.s.d. in the dihedral angle between two l.s. planes) are estimated using the full covariance matrix. The cell e.s.d.'s are taken into account individually in the estimation of e.s.d.'s in distances, angles and torsion angles; correlations between e.s.d.'s in cell parameters are only used when they are defined by crystal symmetry. An approximate (isotropic) treatment of cell e.s.d.'s is used for estimating e.s.d.'s involving l.s. planes.
Refinement. Refinement of *F*^2^ against ALL reflections. The weighted *R*-factor *wR* and goodness of fit *S* are based on *F*^2^, conventional *R*-factors *R* are based on *F*, with *F* set to zero for negative *F*^2^. The threshold expression of *F*^2^ > σ(*F*^2^) is used only for calculating *R*-factors(gt) *etc*. and is not relevant to the choice of reflections for refinement. *R*-factors based on *F*^2^ are statistically about twice as large as those based on *F*, and *R*- factors based on ALL data will be even larger.

### Fractional atomic coordinates and isotropic or equivalent isotropic displacement parameters (Å^2^)

**Table:**

	*x*	*y*	*z*	*U*_iso_*/*U*_eq_	
P1	0.89551 (6)	0.42643 (7)	0.69762 (2)	0.02954 (17)	
P2	0.57483 (6)	0.63702 (6)	0.78754 (2)	0.02854 (16)	
O1	0.90841 (18)	0.36241 (19)	0.75328 (6)	0.0396 (4)	
O2	0.9067 (2)	0.6272 (2)	0.69495 (7)	0.0520 (5)	
H2	0.9850	0.6616	0.7184	0.078*	
O3	0.72469 (17)	0.39370 (19)	0.65968 (6)	0.0391 (4)	
H3	0.6783	0.3113	0.6706	0.059*	
O4	1.0255 (2)	0.3435 (3)	0.67058 (7)	0.0600 (5)	
H4	1.1176	0.3437	0.6913	0.090*	
O5	0.69580 (18)	0.48369 (19)	0.80976 (6)	0.0388 (4)	
H5	0.7548	0.4612	0.7886	0.058*	
O6	0.49043 (19)	0.59476 (19)	0.72822 (6)	0.0387 (4)	
H6	0.4372	0.5041	0.7269	0.058*	
O7	0.66900 (17)	0.80396 (18)	0.78532 (7)	0.0400 (4)	
O8	0.44861 (18)	0.63937 (18)	0.82220 (6)	0.0379 (4)	
N1	0.6019 (2)	0.8023 (2)	0.64703 (7)	0.0376 (4)	
H1A	0.5247	0.7406	0.6585	0.056*	
H1B	0.5939	0.9133	0.6558	0.056*	
H1C	0.7036	0.7626	0.6621	0.056*	
C1	0.5743 (3)	0.7867 (3)	0.58854 (9)	0.0389 (5)	
C2	0.4324 (3)	0.7015 (3)	0.56111 (10)	0.0469 (6)	
C3	0.4120 (4)	0.6914 (3)	0.50585 (11)	0.0575 (7)	
H3A	0.3183	0.6356	0.4856	0.069*	
C4	0.5292 (4)	0.7631 (4)	0.48074 (10)	0.0613 (7)	
H4A	0.5136	0.7546	0.4439	0.074*	
C5	0.6679 (4)	0.8466 (3)	0.50942 (10)	0.0555 (7)	
H5A	0.7453	0.8938	0.4917	0.067*	
C6	0.6953 (3)	0.8624 (3)	0.56494 (9)	0.0452 (6)	
C7	0.3079 (4)	0.6215 (4)	0.59107 (12)	0.0626 (7)	
H7A	0.3650	0.5291	0.6136	0.075*	
H7B	0.2783	0.7095	0.6144	0.075*	
C8	0.1485 (4)	0.5490 (5)	0.55760 (15)	0.0829 (10)	
H8A	0.0873	0.6398	0.5362	0.124*	
H8B	0.0812	0.5013	0.5804	0.124*	
H8C	0.1750	0.4594	0.5348	0.124*	
C9	0.8439 (4)	0.9572 (4)	0.59828 (11)	0.0589 (7)	
H9A	0.8011	1.0495	0.6173	0.071*	
H9B	0.9036	0.8768	0.6248	0.071*	
C10	0.9693 (4)	1.0357 (5)	0.56996 (13)	0.0706 (8)	
H10A	1.0219	0.9453	0.5537	0.106*	
H10B	1.0532	1.0977	0.5952	0.106*	
H10C	0.9128	1.1143	0.5429	0.106*	

### Atomic displacement parameters (Å^2^)

**Table:**

	*U*^11^	*U*^22^	*U*^33^	*U*^12^	*U*^13^	*U*^23^
P1	0.0263 (3)	0.0247 (3)	0.0378 (3)	0.00059 (19)	0.0075 (2)	0.0007 (2)
P2	0.0267 (3)	0.0179 (3)	0.0428 (3)	0.00200 (18)	0.0113 (2)	0.0022 (2)
O1	0.0415 (8)	0.0350 (8)	0.0435 (8)	0.0127 (6)	0.0116 (7)	0.0082 (6)
O2	0.0617 (11)	0.0258 (9)	0.0574 (11)	−0.0121 (7)	−0.0108 (8)	0.0056 (7)
O3	0.0319 (8)	0.0319 (8)	0.0508 (9)	−0.0057 (6)	0.0033 (7)	0.0076 (7)
O4	0.0376 (9)	0.0916 (15)	0.0514 (10)	0.0181 (9)	0.0113 (7)	−0.0113 (10)
O5	0.0412 (8)	0.0293 (8)	0.0496 (9)	0.0141 (6)	0.0182 (7)	0.0093 (7)
O6	0.0469 (9)	0.0261 (8)	0.0437 (8)	−0.0069 (6)	0.0111 (7)	0.0043 (6)
O7	0.0295 (7)	0.0216 (7)	0.0689 (10)	−0.0021 (6)	0.0105 (7)	0.0031 (7)
O8	0.0375 (8)	0.0277 (8)	0.0533 (9)	0.0065 (6)	0.0203 (7)	0.0026 (7)
N1	0.0473 (10)	0.0293 (9)	0.0390 (10)	0.0005 (8)	0.0154 (8)	0.0022 (7)
C1	0.0564 (13)	0.0247 (10)	0.0368 (11)	0.0084 (9)	0.0130 (10)	0.0011 (8)
C2	0.0609 (14)	0.0301 (12)	0.0472 (13)	0.0067 (11)	0.0065 (11)	0.0013 (10)
C3	0.0760 (18)	0.0397 (14)	0.0497 (15)	0.0056 (13)	−0.0014 (13)	−0.0045 (11)
C4	0.101 (2)	0.0459 (15)	0.0362 (13)	0.0114 (15)	0.0134 (14)	−0.0023 (11)
C5	0.0851 (19)	0.0433 (14)	0.0443 (14)	0.0056 (13)	0.0279 (13)	0.0005 (11)
C6	0.0645 (15)	0.0335 (12)	0.0421 (12)	0.0054 (10)	0.0215 (11)	0.0010 (10)
C7	0.0640 (17)	0.0617 (18)	0.0573 (16)	−0.0121 (14)	0.0027 (13)	0.0002 (13)
C8	0.074 (2)	0.079 (2)	0.089 (2)	−0.0143 (18)	0.0033 (18)	−0.0070 (19)
C9	0.0748 (18)	0.0567 (16)	0.0522 (15)	−0.0140 (14)	0.0290 (13)	−0.0032 (12)
C10	0.077 (2)	0.077 (2)	0.0653 (18)	−0.0115 (17)	0.0303 (15)	0.0042 (16)

### Geometric parameters (Å, °)

**Table:**

P1—O1	1.4937 (16)	C2—C7	1.532 (4)
P1—O4	1.5296 (16)	C3—C4	1.381 (4)
P1—O3	1.5364 (15)	C3—H3A	0.9300
P1—O2	1.5525 (17)	C4—C5	1.368 (4)
P2—O8	1.5022 (15)	C4—H4A	0.9300
P2—O7	1.5063 (15)	C5—C6	1.399 (3)
P2—O6	1.5622 (16)	C5—H5A	0.9300
P2—O5	1.5652 (14)	C6—C9	1.511 (4)
O2—H2	0.8200	C7—C8	1.501 (4)
O3—H3	0.8200	C7—H7A	0.9700
O4—H4	0.8200	C7—H7B	0.9700
O5—H5	0.8200	C8—H8A	0.9600
O6—H6	0.8200	C8—H8B	0.9600
N1—C1	1.474 (3)	C8—H8C	0.9600
N1—H1A	0.8900	C9—C10	1.507 (4)
N1—H1B	0.8900	C9—H9A	0.9700
N1—H1C	0.8900	C9—H9B	0.9700
C1—C2	1.382 (3)	C10—H10A	0.9600
C1—C6	1.395 (3)	C10—H10B	0.9600
C2—C3	1.394 (4)	C10—H10C	0.9600
			
O1—P1—O4	112.72 (10)	C5—C4—H4A	119.6
O1—P1—O3	114.58 (9)	C3—C4—H4A	119.6
O4—P1—O3	105.49 (10)	C4—C5—C6	121.2 (3)
O1—P1—O2	112.27 (10)	C4—C5—H5A	119.4
O4—P1—O2	110.04 (12)	C6—C5—H5A	119.4
O3—P1—O2	100.93 (9)	C1—C6—C5	115.9 (2)
O8—P2—O7	115.74 (9)	C1—C6—C9	120.9 (2)
O8—P2—O6	111.54 (9)	C5—C6—C9	123.2 (2)
O7—P2—O6	105.11 (9)	C8—C7—C2	116.7 (3)
O8—P2—O5	104.66 (8)	C8—C7—H7A	108.1
O7—P2—O5	111.86 (9)	C2—C7—H7A	108.1
O6—P2—O5	107.82 (9)	C8—C7—H7B	108.1
P1—O2—H2	109.5	C2—C7—H7B	108.1
P1—O3—H3	109.5	H7A—C7—H7B	107.3
P1—O4—H4	109.5	C7—C8—H8A	109.5
P2—O5—H5	109.5	C7—C8—H8B	109.5
P2—O6—H6	109.5	H8A—C8—H8B	109.5
C1—N1—H1A	109.5	C7—C8—H8C	109.5
C1—N1—H1B	109.5	H8A—C8—H8C	109.5
H1A—N1—H1B	109.5	H8B—C8—H8C	109.5
C1—N1—H1C	109.5	C10—C9—C6	117.8 (2)
H1A—N1—H1C	109.5	C10—C9—H9A	107.8
H1B—N1—H1C	109.5	C6—C9—H9A	107.8
C2—C1—C6	124.9 (2)	C10—C9—H9B	107.8
C2—C1—N1	118.8 (2)	C6—C9—H9B	107.8
C6—C1—N1	116.4 (2)	H9A—C9—H9B	107.2
C1—C2—C3	116.4 (2)	C9—C10—H10A	109.5
C1—C2—C7	120.6 (2)	C9—C10—H10B	109.5
C3—C2—C7	123.0 (2)	H10A—C10—H10B	109.5
C4—C3—C2	120.8 (3)	C9—C10—H10C	109.5
C4—C3—H3A	119.6	H10A—C10—H10C	109.5
C2—C3—H3A	119.6	H10B—C10—H10C	109.5
C5—C4—C3	120.9 (2)		

### Hydrogen-bond geometry (Å, °)

**Table:**

*D*—H···*A*	*D*—H	H···*A*	*D*···*A*	*D*—H···*A*
O2—H2···O1^i^	0.82	1.84	2.540 (2)	142.
O3—H3···O8^ii^	0.82	1.72	2.520 (2)	166.
O4—H4···O7^iii^	0.82	1.74	2.521 (2)	158.
O5—H5···O1	0.82	1.86	2.664 (2)	165.
O6—H6···O7^ii^	0.82	1.76	2.577 (2)	171.
N1—H1A···O6	0.89	2.18	2.927 (2)	141.
N1—H1B···O8^iv^	0.89	1.89	2.772 (2)	172.
N1—H1C···O2	0.89	1.98	2.861 (3)	168.

Symmetry codes: (i) −*x*+2, *y*+1/2, −*z*+3/2; (ii) −*x*+1, *y*−1/2, −*z*+3/2; (iii) −*x*+2, *y*−1/2, −*z*+3/2; (iv) −*x*+1, *y*+1/2, −*z*+3/2.
